# GPR Feature Enhancement of Asphalt Pavement Hidden Defects Using Computational-Efficient Image Processing Techniques

**DOI:** 10.3390/ma18184400

**Published:** 2025-09-20

**Authors:** Shengjia Xie, Jingsong Chen, Ming Cai, Zhiqiang Cheng, Siqi Wang, Yixiang Zhang

**Affiliations:** 1 Shanghai Road and Bridge Group Co., Ltd., Shanghai 200433, China; sjxie0518@163.com (S.X.); 13918535375@163.com (M.C.); cr420281@163.com (Z.C.); 2 Shanghai Engineering Research Center of Green Pavement Materials, Shanghai 200433, China; 3 YueXiu (China) Transport Infrastructure Investment Limited, Guangzhou 510000, China; chenjs923@163.com; 4 School of Transportation, Southeast University, Nanjing 211189, China; 220233392@seu.edu.cn

**Keywords:** ground-penetrating radar, asphalt pavement, image processing, non-destructive testing

## Abstract

Hyperbolic reflection features from ground-penetrating radar (GPR) data have been recognized as essential indicators for detecting hidden defects in the asphalt pavement. Computer vision and deep learning algorithms have been developed to detect and enhance the hyperbolic features of hidden defects. However, migrating existing hyperbolic feature detection methods using raw GPR data results in inaccurate predictions. Pre-processing raw GPR data using straightforward image processing methods could enhance the reflection features for fast and accurate hyperbolic detection during real-time GPR measurements. This study proposed accessible and straightforward image processing methods as GPR data preprocessing steps (such as the Sobel edge detector and histogram equalization) to assist existing computer vision algorithms for reflection feature enhancement during the GPR survey. Field tests were conducted, and several image processing methods with existing standard image processing libraries were applied. The proposed regions of the identified hyperbola signal-to-noise ratio (RIHSNR) were used to quantify the enhancement performance of hyperbolic feature detectability. Applying Sobel edge detection and Otsu’s thresholding to GPR data significantly improves detection accuracy and speed: mAP@0.5 rises from 0.65 to 0.85 for Faster R-CNN and from 0.72 to 0.88 for CBAM-YOLOv8 using the proposed computer vision methods as preprocessing steps. At the same time, inference time drops to 30 ms and 25 ms, respectively.

## 1. Introduction

Ground-penetrating radar (GPR) has been implemented for hidden defects detection in asphalt pavements [1,2]. It sends and receives electromagnetic (EM) signals, i.e., traces or A-scans, and generates two-dimensional images as a distorted mapping of the subsurface conditions, i.e., radargrams or B-scan [3,4,5]. Stratified or hyperbolic features in the obtained images are indicators of layered and localized objects, respectively, which can be identified via visual inspection during the GPR survey [6,7,8]. Hyperbolic feature enhancement using GPR data has been recognized as a key indicator for hidden defect detection for pavements [9,10,11]. Weak reflections are challenging to unveil using low signal-to-noise (SNR) GPR data [12]. Attributes such as edge histogram [13], statistical parameters [14], change of EM wave propagation velocity [15], and multi-frequency features [16] have been used for hidden defect detection. Pattern recognition and computer vision methods are implemented for hyperbolic feature detection [17,18,19].

Hyperbolic reflection features are usually weak due to the low SNR in the GPR collected from the asphalt pavement. Noise can be attenuated using background removal methods [20] and frequency-domain features [21]. Advanced machine learning- and deep learning-based algorithms such as convolutional neural networks (CNNs), support vector machines (SVMs), hidden Markov models (HMMs), genetic algorithms, and other methods are applied for noise cancellation in GPR applications [22,23,24]. Despite high detection accuracy, these methods require multiple processing steps, complicated structures, and hyperparameter setups. Existing computer vision algorithms, such as C3 clustering, DSCE algorithm, and deep learning approaches, have been developed and implemented for hyperbolic feature detection using GPR [25,26].

Two-stage detectors (e.g., Faster R-CNN) achieve high precision via cascaded proposal and recognition. Pham first localized GPR hyperbolae, Cui then detected highway interfaces [27], Zhao segmented tunnel-lining defects [28], and Hou/Huang improved complex target recognition with custom anchors, self-attention, and dense contrastive learning [29,30]. Yet cascaded errors, long training, and weak fusion still raise false alarms. Single-stage SSD/YOLO families favor real-time underground tasks. Wang boosted SSD with FPN and GIoU [31], Qiu/Wu refined YOLOv5 for small objects [32,33], Hu added attention for hyperbolae [34], and Wang embedded CBAM in YOLOv8 for urban defects [35]. All, however, rely heavily on costly, high-quality annotations in complex multi-physics GPR scenes.

To automate GPR-based pavement inspection, complementary pipelines have been proposed. A YOLOv3–U-Net cascade first detects distress echoes with 96.99% overall accuracy, then segments pixels (mIoU ≥ 0.856), and finally fits hyperbola centroids to localize cracks or debonding within a 3.25 cm depth error [36]. For void assessment, CWT-enhanced 3D time–frequency imaging highlights energy concentrations in reconstructed B-scans, enabling precise width and depth extraction validated in both FDTD and laboratory models [37]. Meanwhile, an improved YOLOv8—augmented by AutoAugment, a 160 × 160 output layer, SKNet attention, and Power-IoU loss—achieves 90.7% mAP and 90.1% F1 for crack hyperbola detection, outperforming baseline YOLOv8 by 6.3% and 5.9%, respectively, and matching coring results in field surveys [38].

The above approaches show that migrating existing algorithms to raw GPR data in a new testing environment could result in poor detection performance. GPR signal processing using built-in functions and/or algorithms of standard image processing libraries could be efficient for operators in identifying hyperbolas during GPR field surveys. Combining image processing methods as preprocessing and as complements for existing computer vision methods could enable fast and accurate hyperbolic feature detection using GPR, particularly in complex testing environments with low signal-to-noise ratios. Hence, while deep learning methods have advanced hyperbola detection in GPR data, challenges remain regarding data requirements, labeling efforts, and model adaptability to complex underground environments. Using straightforward image processing methods as complements for existing hyperbolic feature detection algorithms could significantly enhance efficiency and accuracy and reduce dependency on extensive labeled data.

This study proposed accessible and straightforward image processing methods to assist feature enhancement of hidden defects in the asphalt pavement using GPR. This could accelerate the visualization of hidden defects to assist efficient maintenance and rehabilitation operations. Factors that may affect the detectability of the hyperbolic features (intensity, shape, and location) in the asphalt pavement using GPR were discussed. Field tests were conducted. Several image processing methods, such as edge detection, thresholding, histogram equalization, and clustering, were applied to field test GPR data. The region of identified hyperbola signal-to-noise ratio (RIHSNR) was proposed to quantify the performance of hyperbolic feature enhancement. The optimal input parameters of these methods were discussed based on RIHSNR results. The suggested combination of image processing methods and existing computer vision algorithms may serve as real-time processing steps to enhance the detectability of hyperbolas during GPR surveys. This could help GPR operators to identify the hidden defects during real-time GPR measurements.

## 2. Field Test

### 2.1. Raw Material Properties

#### 2.1.1. Asphalt Binder

In this study, a high-viscosity asphalt binder and a heavy-duty asphalt binder were employed. Their key technical properties were evaluated following the Test Methods of Bitumen and Bituminous Mixtures for Highway Engineering. The corresponding test results are presented in Table 1.

#### 2.1.2. Aggregate

Aggregates constitute a fundamental constituent of asphalt mixtures, and their mechanical characteristics are among the primary determinants of mixture strength [39,40]. Particle morphology governs the aggregate skeleton architecture, thereby directly influencing rutting resistance and fatigue performance. Moreover, the adhesion quality at the aggregate–binder interface dictates moisture damage resistance and long-term durability. Consequently, the utilization of high-quality aggregate is an essential prerequisite for superior pavement performance. The relevant aggregate densities are reported in Table 2.

#### 2.1.3. Mineral Filler

Mineral filler is the fine material incorporated into asphalt mixtures to occupy micro-voids. In laboratory practice, it is conventionally defined as the fraction passing the 0.075 mm sieve. Functionally, the filler fulfils two primary roles: (i) the physical densification of the mixture by filling interstitial voids, and (ii) the formation of a stiff mastic through intimate interaction with the asphalt binder, thereby enhancing mixture strength and stability. According to prevailing specifications, filler should be derived from the comminution of strong alkaline rocks such as limestone or magmatic rocks. In the present study, a limestone filler was adopted.

#### 2.1.4. Fiber

The incorporation of fibers as stabilizing agents into asphalt mixtures has been demonstrated to markedly enhance the low-temperature crack resistance and high-temperature rutting resistance of asphalt concrete. At present, lignin fibers, mineral fibers, and polymer fibers are the fiber stabilizers most frequently employed in bituminous mixtures. In this investigation, lignin fiber was selected as the stabilizing additive. Its principal mechanism of action rests on the capacity of the dispersed fibers to increase the effective viscosity of the bituminous mastic, thereby ensuring firm and durable adhesion of the binder to the aggregate surfaces. Functioning as a modifier, the lignin fiber substantially improves the ageing resistance of the asphalt, enabling the mixture to sustain prolonged exposure to mechanical loading and environmental temperature variations, and thus extending service life. Consequently, fiber-reinforced stone mastic asphalt (SMA) exhibits markedly enhanced pavement performance. In the laboratory phase reported herein, lignin fiber was incorporated into the SMA mixture at a dosage of 0.3% by mass of the total mixture.

#### 2.1.5. Mixture Design

In accordance with the Technical Specifications for Construction of Highway Asphalt Pavements and practical engineering experience, three asphalt mixture gradation types—AC-20, SMA-10, and SMA-13—were adopted. For SMA-13, three gradation variants (coarse, medium, and fine) were investigated, with the passing percentages at the 4.75 mm sieve set at 25.0%, 27.6%, and 30.3%, respectively. The VCA_DRC_ (voids in coarse aggregate in a dry rodded condition) was determined for each gradation. An initial asphalt–aggregate ratio of 5.8% was employed, and Marshall specimens were fabricated by applying 75 blows on each side. Marshall tests were conducted to obtain VCAmix (voids in coarse aggregate of the compacted mix) and VMA (voids in mineral aggregate). The final design gradation was selected based on the simultaneous fulfillment of VCAmix < VCA_DRC_ and VMA > 17%.

Based on the three finalized optimal gradations, standard Marshall specimens for four asphalt mixtures were fabricated in strict accordance with the relevant specifications. Marshall testing was subsequently conducted to determine the respective volumetric and mechanical indices for each mixture, thereby establishing the optimum asphalt–aggregate ratio for all four mixtures, as reported in Table 3.

### 2.2. Field Test

**Figure 1:**
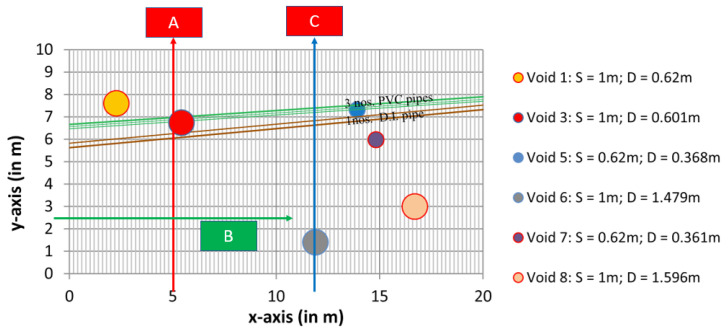
Survey lines at the field test site. Two survey lines are highlighted. Data from Line A was processed in this study.

**Figure 2:**
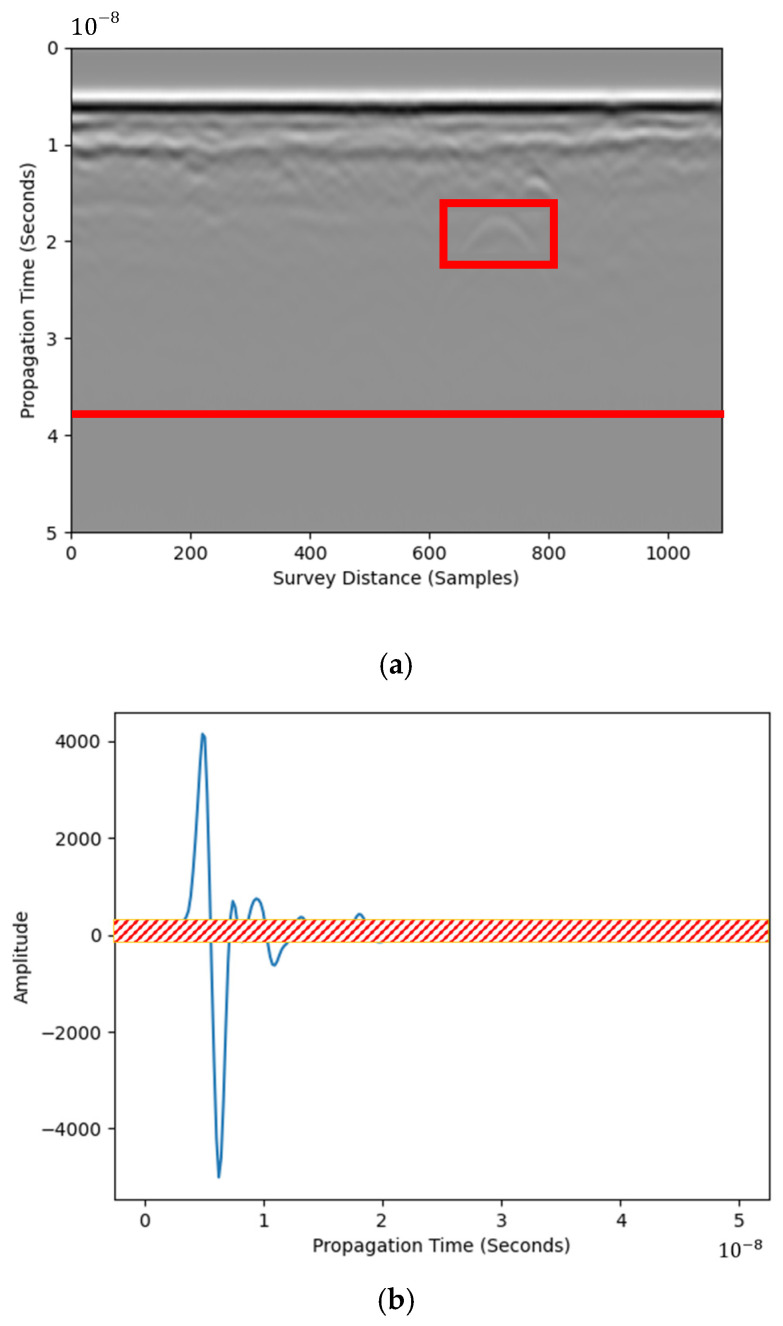
B-scan image from field test data: (**a**) highlighted hyperbola and pure noise boundary and (**b**) corresponding A-scan signal from field test data with the demonstrated noise (The red box is the detection target).

Field tests were performed in an asphalt pavement section. Plastic blocks were prefabricated and embedded in the pavement layer to simulate air cavities, which are referred to as voids in Figure 1. The figure also shows GPR survey lines. Both 1.5 GHz and 1 GHz ground-coupled GPR antennas from IDS were used in the test. The GPR B-scan image from survey line A (shown in Figure 1) was used for raw data. The plastic box reflection is shown as the highlighted hyperbola in Figure 2a.

### 2.3. Region of Identified Hyperbola Signal-to-Noise Ratio (RIHSNR)

Figure 2b shows an example of a field-obtained A-scan signal extracted from the B-scan image in Figure 2a. Reflection from the target is partially camouflaged by noise, as shown in the red shadow. RIHSNR was proposed to quantify the visibility of hyperbolic reflection features using Figure 2a,b as examples—the hyperbola is visible if the maximum amplitude of the highlighted area is larger than the background noise level. Compared with traditional SNR, RIHSNR was calculated by estimating the SNR after first framing, which is more targeted than the traditional SNR index calculation based on the whole image domain. Noise level is obtained using the mean absolute amplitude after 39 ns, as shown in the red horizontal line in Figure 2a. The GPR signal attenuates after 39 ns, where no pattern is observed. Note that this is an example for analyzing the RIHSNR by identifying 39 ns. Hence, RIHSNR ($R_{RIH}$) is defined using the following Equation:

(1) $$R_{RIH}=\frac{Abs(Max\left(A_{roi}\right))}{Abs(Mean\left(A_{noise}\right))}$$

where Abs, Max, and Mean are absolute value, maximum value, and mean value operators, respectively; $A_{roi}$ is the pixel value in the identified hyperbola area, as shown in the rectangular highlight in Figure 2a; and $A_{noise}$ is the pixel value in the noise region, as shown beneath the red highlight line in Figure 2a. An RIHSNR score using the image processing method, higher than the raw B-scan image, indicates enhancement of hyperbola visibility. The RIHSNR score of the raw B-scan image in Figure 2a is 5.83. RIHSNR values may vary if a different identified hyperbola region is selected. However, the change of RIHSNR using a specific method is independent of the identified hyperbola region.

## 3. Image Processing of Raw GPR Radargrams for Feature Enhancement

Functions from existing standard image processing libraries can enhance the visibility of hyperbolic reflection features in the B-scan image, allowing operators to make informed engineering judgments quickly in the field. In this study, the Python 3.11 libraries were utilized.

### 3.1. Edge Detection Approaches

**Figure 3:**
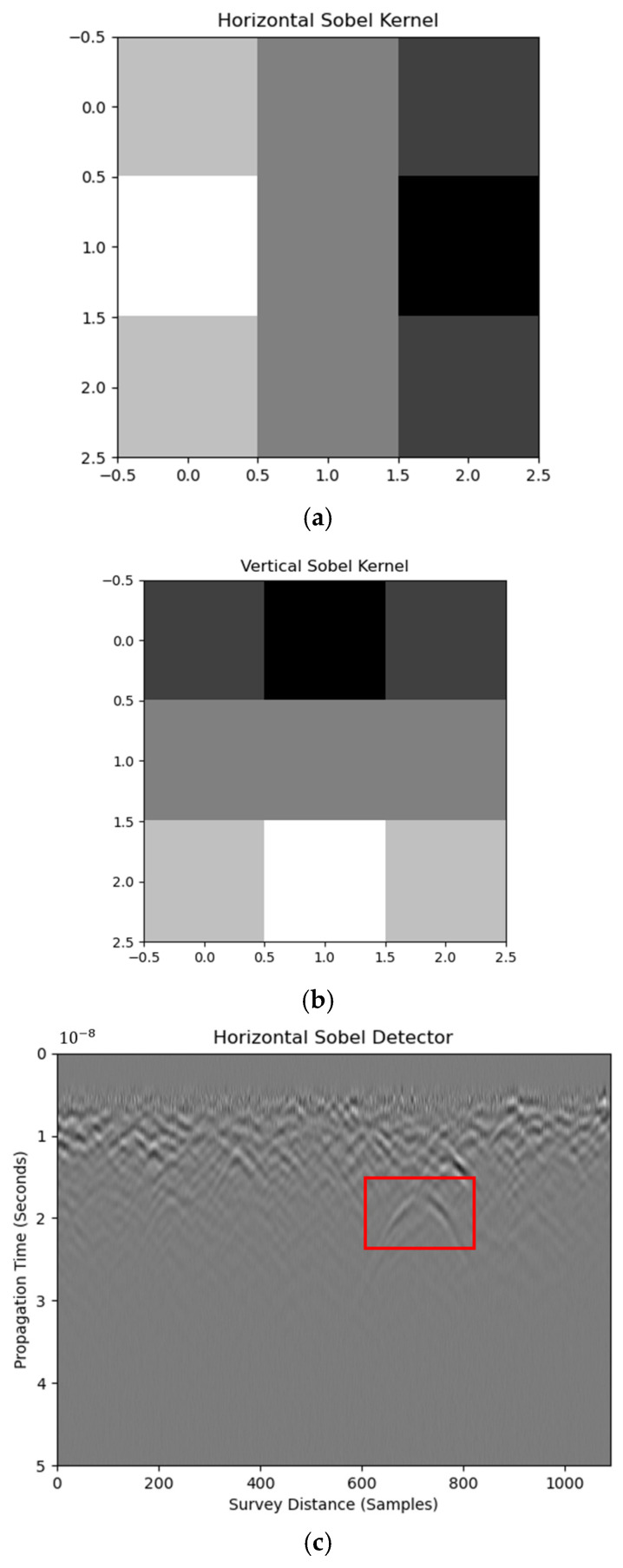

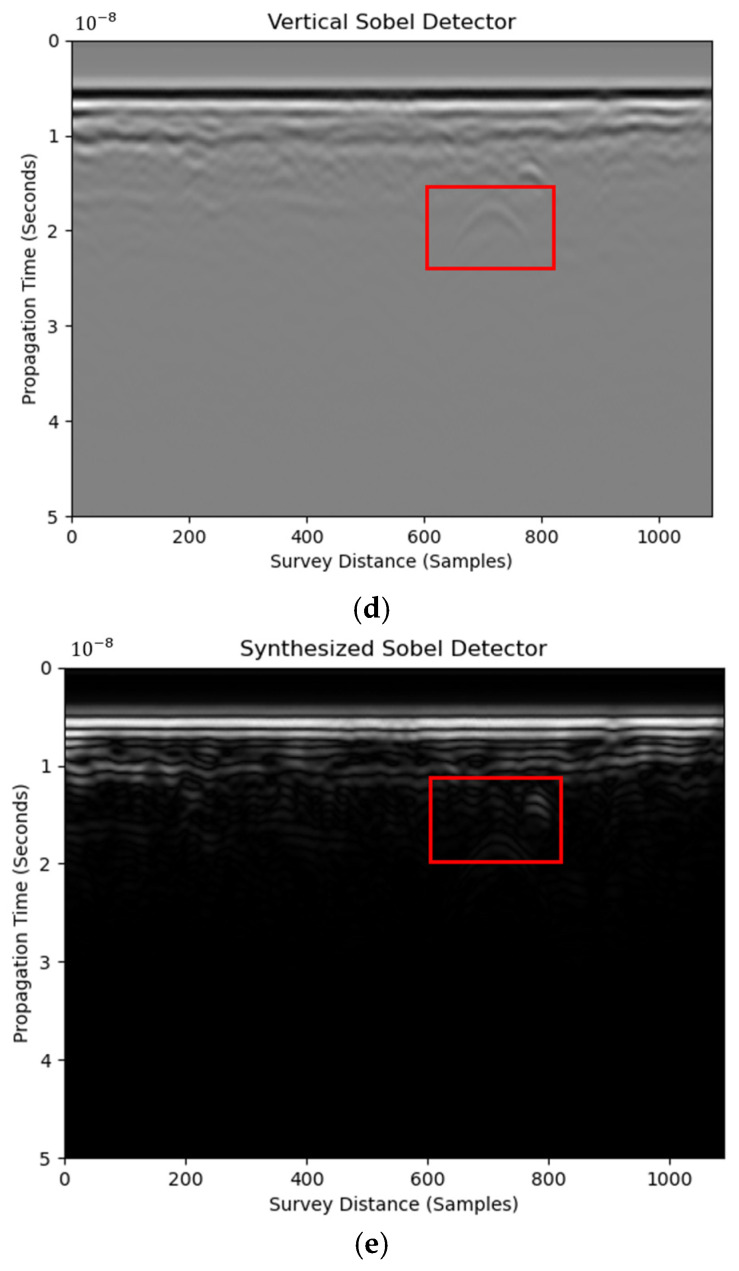
Horizontal (**a**) and vertical Sobel kernels (**b**) used to process B-scan image as horizontal Sobel (**c**), vertical Sobel (**d**), and synthesized Sobel results (**e**). (The red box is the detection target. The images below are the same.)

Edge-enhancement operators aim to emphasize boundaries characterized by steep gray-level gradients. In a B-scan, each pixel’s value is essentially a voltage scaled by a manufacturer-supplied calibration factor, so edge information is unaffected by vendor-specific gain functions. Among the convolution-based detectors, the 3 × 3 Sobel kernel is the most widely adopted; its response is binarized via Otsu’s adaptive threshold, as detailed in Equations (2)–(5):

(2) $$L_{x}=\left[\begin{array}{ccc} 1 & 0 & -1\\ 2 & 0 & -2\\ 1 & 0 & -1 \end{array}\right]$$

(3) $$L_{y}=\left[\begin{array}{ccc} 1 & 2 & 1\\ 0 & 0 & 0\\ -1 & -2 & -1 \end{array}\right]$$

(4) $$\left|\nabla L\right|=\sqrt{L_{x}^{2}+L_{y}^{2}}$$

(5) $$\theta =\tan^{-1}\left(L_{y},L_{x}\right)$$

where $L_{x}$ and $L_{y}$ are the gradient extractors at horizontal and vertical directions, respectively and ∇L and θ are the norm and orientation of the synthesized gradient, respectively. Equations (2) and (3) are respective horizontal and vertical Sobel kernels that extract edges at corresponding directions, as shown in Figure 3a,b. The geometric mean of the two kernel responses, or synthesized Sobel kernel, is calculated to provide the edge features of the raw image, as shown in Figure 3c,d. Edge features are obtained by convolving the raw image with kernels, shown in Equation (6), as follows:

(6) $$g\left[m,n\right]=\left(f\times k\right)\left[m,n\right]=\sum_{i,j}f\left[m-i,n-j\right]k\left[i,j\right]$$

where $g\left[m,n\right]$, $f\left[m,n\right]$ and $k\left[m,n\right]$ are the edge map, raw image, and the kernel, respectively. 

The horizontal Sobel kernel captures hyperbolic features only, as shown in Figure 3b. Stratified patterns caused by layer reflection are excluded. This indicates the effectiveness of the horizontal Sobel detector in unveiling localized weak reflections, such as plastic boxes in this case. In addition, the RIHSNR score after the horizontal Sobel detector is 30.92, which is higher than that of the raw image (5.83), showing the visibility enhancement of hyperbolic features. Meanwhile, enhancement using a synthesized Sobel kernel is less effective than the horizontal one. Hence, it is suggested to use the horizontal Sobel detector to process raw B-scan images for hyperbolic reflection enhancement during the GPR survey. It is worth mentioning that the Sobel kernel had comparable performance to the Gaussian and median kernels, which have better computational efficiency, as documented in [17].

### 3.2. Thresholding Approaches

The thresholding operation divides the image into two segments: foreground and background, enhancing certain features. Hyperbolas are identified as the foreground with pixel values different from the background. Binary thresholding sets a global pixel value for the entire image, which could vary depending on the operator’s experience in image processing. Otsu’s thresholding performs binary thresholding using the intra-class correlation between the image’s foreground and background. The global threshold is identified based on the minimization of intra-class variance or maximization of inter-class variance between the foreground and background, as shown in the following Equation (7):

(7) $$\sigma_{w}^{2}=\omega_{0}\left(t\right)\sigma_{0}^{2}\left(t\right)+\omega_{1}\left(t\right)\sigma_{1}^{2}\left(t\right)$$

where $\sigma_{w}^{2}$ is the intra-class variance between foreground and background; $\omega_{0}$ and $\omega_{1}$ are the probabilities of foreground and background, respectively, separated by the global threshold pixel value *t*; and $\sigma_{0}^{2}\left(t\right)$ and $\sigma_{1}^{2}\left(t\right)$ are the variances of foreground and background, respectively.

**Figure 4:**
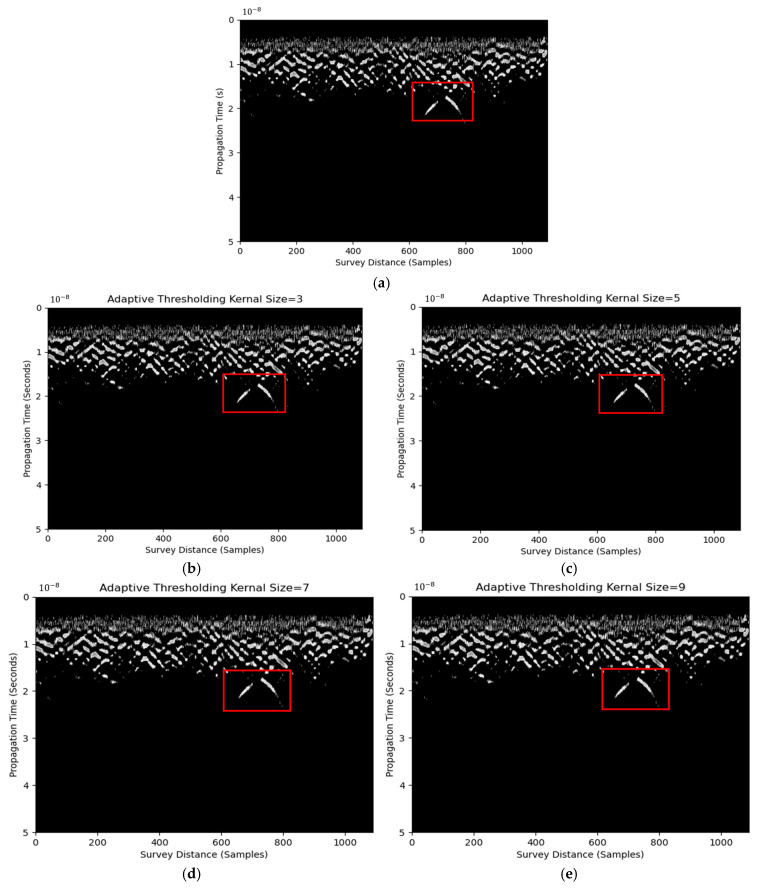
B-scan image using Otsu’s thresholding (**a**) and adaptive thresholding using kernel size of three (**b**), five (**c**), seven (**d**), and nine (**e**).

The horizontal Sobel edge detector was applied before Otsu’s thresholding. Pixel values less than this threshold were set as background noise, as shown in Figure 4a. The target hyperbola was more evident and separated from the surroundings than that using solely the horizontal Sobel detector in Figure 3. This indicates the feasibility of a horizontal Sobel detector and thresholding in enhancing the visibility of hyperbolic reflections.

One major drawback of Otsu’s thresholding is the use of one global threshold, which may enhance target hyperbolas and noise. Adaptive thresholding may address this issue by identifying the threshold based on the region of the pixel-of-interest’s proximity. Thresholds are adjusted for different regions in an image. This could provide a more robust thresholding result when compared with binary thresholding. However, it requires the mean or Gaussian kernel size as an input. Results using kernel sizes of three, five, seven, and nine are shown in Figure 4. Minimal improvements can be observed among different kernel sizes. RIHSNR scores are summarized in Table 4. Despite the improvement of hyperbola visibility with the increase in kernel size, all of them are higher than the raw image RIHSNR score. The images differ from the edge detector results in Figure 2b by visual inspection because only black and white pixels are displayed after thresholding. Hence, a default adaptive thresholding kernel size of three or five, or simply Otsu’s thresholding, is suggested after the horizontal Sobel detector for hyperbolic feature enhancement.

### 3.3. Histogram Equalization

**Figure 5:**
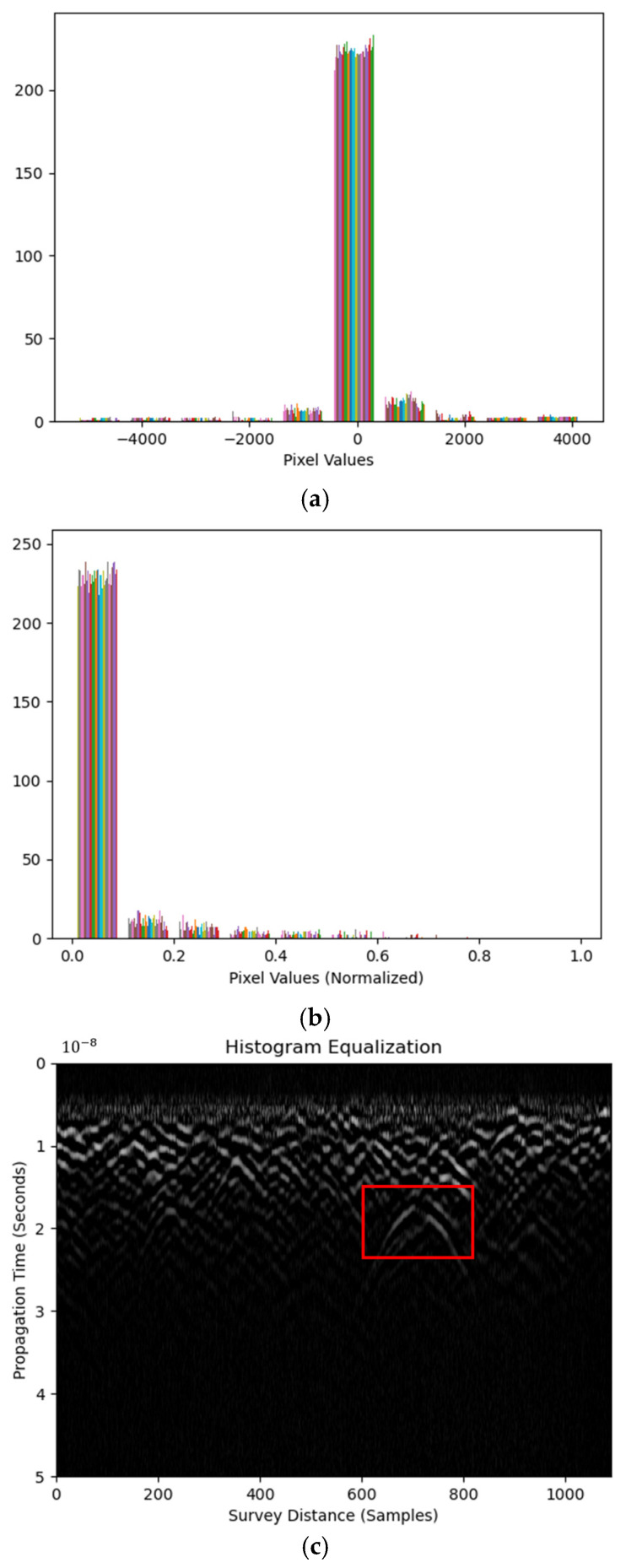
Histograms of B-scan image (**a**) after equalization operation and (**b**) after CLAHE. (**c**) The processed B-scan image.

Image histogram is a probability distribution function (PDF) over the pixel values. Heights in the histogram represent pixel counts within corresponding bins. Figure 5a is the histogram from the raw B-scan image. It shows most of the image contrast information located at pixel values from −1000 to 1000, indicating limited values as the feature pixels.

Equalization transforms the histogram to be more uniformly distributed across all bins than the original image. This is because the image contrast is enhanced if the histogram ‘covers’ more pixel values, i.e., the PDF of the pixel values is distributed more evenly. It is operated using Equations (8)–(11), as follows:

(8) $$p_{x}\left(i\right)=p\left(x=i\right)=\frac{n_{i}}{N}$$

(9) $${CDF}_{x}\left(i\right)=\sum_{j=0}^{i}p_{x}\left(x=j\right)$$

(10) $$y=Trans\left(k\right)={CDF}_{x}\left(k\right)$$

(11) $${CDF}_{y}\left(i\right)=\left(i+1\right)K$$

where $p_{x}\left(i\right)$ is the PDF of the raw image x with pixel value of i counts of $n_{i}$; ${CDF}_{x}\left(i\right)$ and ${CDF}_{y}\left(i\right)$ are the cumulative density functions (CDFs) of image *x* and *y*, respectively, which are before and after histogram equalization; and $Trans\left(k\right)$ is a transformation function that produces a linear ${CDF}_{y}\left(i\right)$ using ${CDF}_{x}\left(i\right)$. *K* is defined as the reciprocal of the total number of pixels in the image, i.e., *K* = 1/*N*, where *N* is the total pixel count.

An adaptive histogram equalization (AHE) method processes histogram equalization in localized fractions instead of the whole image. This may avoid enhancing the noise level along with the image contrast at the full image scale. It is suggested that AHE be applied using the contrast limited AHE (CLAHE) method in engineering practices. A clip limit is used to limit the histogram before calculating the CDF for equalization. This suppresses the CDF slope to provide a more uniformly distributed histogram after AHE. Image pixel values are normalized before CLAHE. Figure 5b shows the histogram using CLAHE with a clip limit of 0.01, which is the default value. Note that CLAHE was performed after horizontal Sobel edge detection. Large portions of pixel values are still accumulated, contributing to limited hyperbolic feature visibility enhancement, as shown in Figure 5c. RIHSNR scores using various clip limit values are shown in Table 5. It is concluded that histogram equalization, even using the horizontal Sobel detector processed B-scan, is not as effective as thresholding in hyperbolic feature enhancement.

### 3.4. Clustering Approach

**Figure 6:**
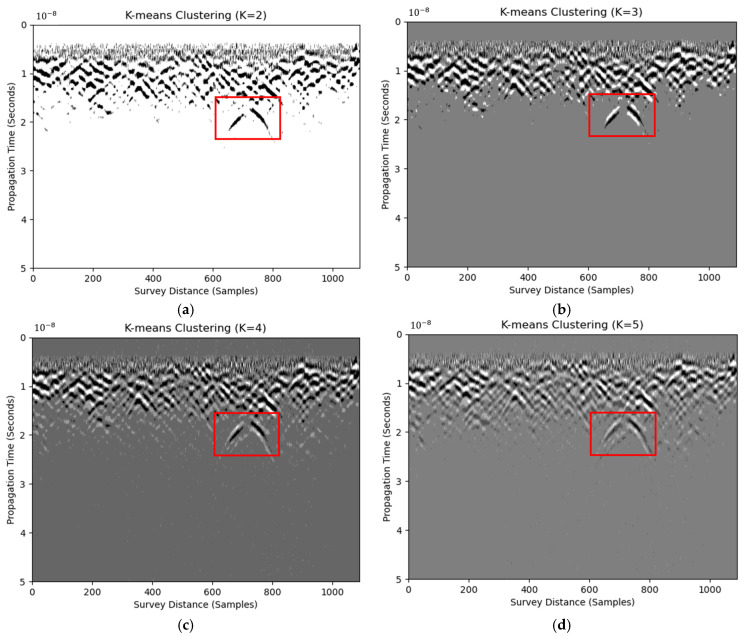
B-scan images using K-means clustering with cluster numbers of two (**a**), three (**b**), four (**c**), and five (**d**). Red boxes are regions of detected defects.

Clustering is an unsupervised machine learning paradigm that groups pixels (or higher-level image descriptors) into coherent, non-overlapping subsets without external labels. The hyperbolic reflection features consist of the cluster(s) in the radargram image. The Euclidean distance, or L-2 norm, is used to calculate the ‘similarity’ among image segments, as shown in the following Equation (12):

(12) $$\underset{S}{\mathrm{argmin}}\left\{\sum_{i=1}^{k}\sum_{X\in S_{i}}{\left\|X-\mu_{i}\right\|}^{2}\right\}$$

where X are the observations, which are the pixel values in the radargram image; S are the clusters after K-means clustering; and $\mu_{i}$ are the means of points within cluster $S_{i}$. Equation (12) requires the input K to be the cluster number. The results of clustering using K from two to five are shown in Figure 6. Note that RIHSNR scores may alter because K-means clustering identifies different centroids randomly in each execution. For example, the clustered results transitioned from white scale to black scale when changing K from 2 to 3. This indicates the lack of robustness of the clustering method. To mitigate the abrupt RIHSNR fluctuations caused by increasing K, a within-image normalization is applied to every clustered map before the score is computed. Immediately after K-means converged, the pixel-wise label image L was converted into a pseudo-gray-level image G. This operation compressed the dynamic range of G to the fixed interval [0, 1], regardless of the chosen K. This ensures the subsequent RIHSNR evaluation to be comparable to the original radargram, with a normalized label map whose contrast does not scale with K. RIHSNR scores are shown in Table 6. In conclusion, even the use of normalized clustering in processed B-scans is not as effective as thresholding in enhancing hyperbolic features.

In summary, several image processing methods were applied to the field test data. The RIHSNR score results suggest using a horizontal Sobel edge detector, adaptive thresholding using default kernel size, or Otsu’s thresholding during GPR data collection.

## 4. Impact of Image Processing on Deep Learning for GPR-Based Defect Detection

In GPR image processing and object detection, combining image processing with deep learning can significantly improve the efficiency and accuracy of detection. GPR images often have low signal-to-noise ratios, complex backgrounds, and unclear target features, making direct deep learning applications less effective. Preprocessing GPR images with image processing methods can enhance target features and reduce noise, improving the accuracy and reliability of subsequent object detection.

Faster R-CNN and YOLOv8 have been widely used deep learning algorithms in object detection. Faster R-CNN, a two-stage model with a region proposal network (RPN), improves detection speed and accuracy. YOLOv8, a single-stage model, emphasizes real-time performance and high frame rates. Combining image processing with these two algorithms can leverage the advantages of image processing in feature enhancement and noise suppression, further improving detection performance. This section compares whether processed images enhance recognition accuracy by comparing processed and raw images from Section 3 for object detection with Faster R-CNN and YOLOv8.

### 4.1. Field Test Results

The primary objective of this experiment is to evaluate whether the image preprocessing methods proposed in Section 3 can improve the detection accuracy of deep learning models (Faster R-CNN and Convolutional Block Attention Module (CBAM)-YOLOv8) on GPR data. By comparing the detection results of raw and processed datasets, we aim to determine the effectiveness of the preprocessing techniques in enhancing the performance of these models.

The objective of this field test is to evaluate whether the image preprocessing methods proposed in Section 3 can improve the detection accuracy of deep learning models (Faster R-CNN and Convolutional Block Attention Module (CBAM)-YOLOv8) on GPR data. By comparing the detection results of raw and processed datasets, we aim to determine the effectiveness of the preprocessing techniques in enhancing the performance of these models. All training and inference were performed on a workstation from HP inc. in Shanghai China equipped with an Intel^®^ Core™ i7-14700F CPU (24 cores, 5.8 GHz boost), 32 GB DDR5-5600 RAM, and NVIDIA GeForce RTX 5060 GPU.

The experiment utilizes two datasets for evaluation. The raw dataset consists of original GPR B-scan images collected from field tests, serving as the baseline for comparison. The processed dataset includes images enhanced using the optimal preprocessing method from Section 3, which combines horizontal Sobel edge detection and Otsu’s thresholding to enhance hyperbola features and reduce noise. Both datasets are manually labeled using the open-source software LabelImg 1.8.6 to identify hyperbola regions, ensuring consistent ground truth for target detection across all datasets. This labeling process provides precise bounding box coordinates for the hyperbola regions, facilitating accurate evaluation of the detection models.

In the experimental design, all images are resized to 640 × 640 pixels to ensure dataset consistency and meet the models’ input requirements. A batch size of 16 is chosen to balance training speed and memory usage, ensuring effective gradient computation within hardware constraints. The models are trained for 300 epochs to guarantee convergence and adequate feature learning from the data. For the loss function, a combination of cross-entropy loss and IoU loss is employed; cross-entropy loss evaluates classification accuracy, while IoU loss ensures precise bounding box localization, thus providing a comprehensive assessment of the models’ performance. These settings collectively ensure the stability and efficiency of the training process, laying the foundation for enhancing the accuracy and robustness of object detection.

During the training process, several strategies were employed to optimize model performance. To prevent overfitting and enhance generalization, data augmentation techniques, including random flipping, rotation, and scaling, were applied to the training dataset. For Faster R-CNN, transfer learning was utilized by initializing the model with pre-trained weights from the COCO dataset, which accelerated the training process and improved performance. In contrast, YOLOv8 was initialized with random weights and trained from scratch to ensure it learned features specific to the GPR data. Additionally, key hyperparameters, such as the learning rate, momentum, and weight decay, were carefully tuned. The initial learning rate was set to 0.01, with momentum at 0.937 and weight decay at 0.0005, to optimize model performance. These approaches collectively contributed to the practical training and optimization of the models.

### 4.2. Experiment Workflow

Data Preparation: Both raw and processed datasets are prepared and annotated.

Model Training: Faster R-CNN and CBAM-YOLOv8 are trained separately on both datasets using the specified training configuration.

Performance Evaluation: The trained models are evaluated using the test dataset, and their performance is measured using precision, recall, and mAP.

Result Comparison: The detection results from raw and processed datasets are compared in order to assess the impact of the preprocessing methods on model accuracy.

**Figure 7:**
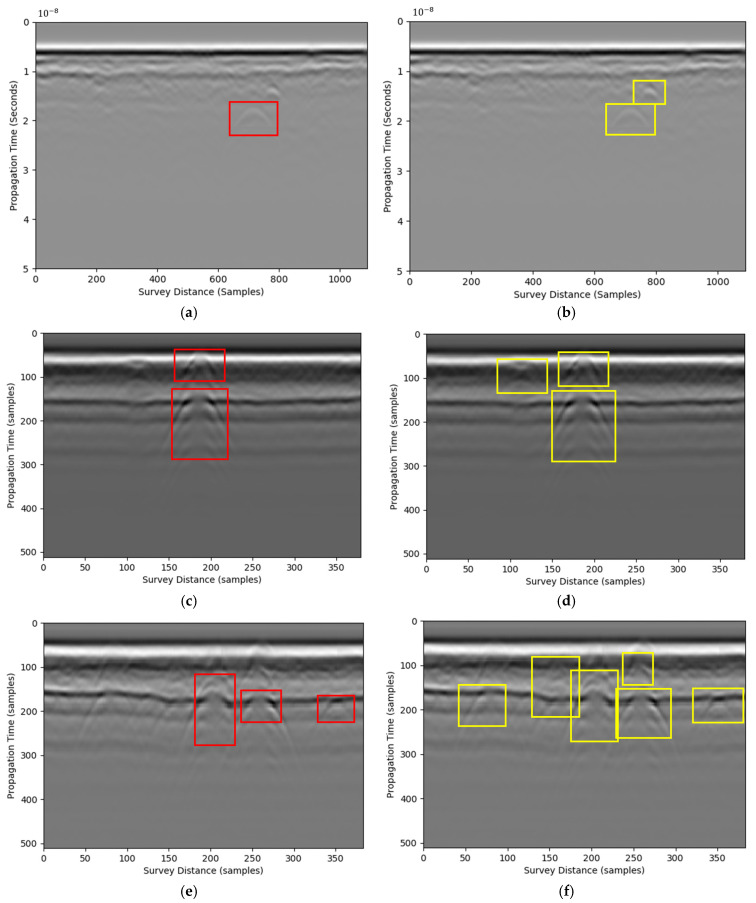
Hyperbolic feature detection results. The red box represents results processed by deep learning without the proposed preprocessing pipeline. The yellow box represents results processed by deep learning using the proposed preprocessing pipeline (**a**,**c**,**e**) are the result before processing, (**b**,**d**,**f**) are the result after processing.

The processed data results are presented in mean average precision (mAP@0.5), F1 score, as well as inference time for processing efficiency and accuracy purposes. Each GPR image was processed for ten repetitive tests to validate the robustness. Results of ten rounds of testing were reported in Table 7. Results show that the processed dataset achieved a higher mAP@0.5 compared with the raw dataset, with Faster R-CNN improving from 0.65 to 0.85 and CBAM-YOLOv8 improving from 0.72 to 0.88. The F1 score, which balances precision and recall, also showed significant gains, increasing from 0.68 to 0.82 for Faster R-CNN and from 0.75 to 0.89 for CBAM-YOLOv8. Additionally, the inference time was reduced, with Faster R-CNN decreasing from 45 ms to 30 ms and CBAM-YOLOv8 decreasing from 30 ms to 25 ms. Note that the preprocessing time is included in the end-to-end timing. The RIHSNR scores are reported in Table 7 using R-CNN and CBAM-YOLOv8 with and without preprocessing using the proposed pipeline. These results demonstrate that integrating image processing with deep learning not only enhances detection accuracy but also improves computational efficiency, making it more suitable for real-time applications in complex environments. Figure 7 shows the detection results. The proposed method could identify more hyperbolic features than simply using the existing approaches. Moreover, this method standardizes image processing, improving the signal-to-noise ratio before image processing and increasing computational efficiency.

The preprocessing techniques employed in this study offer several significant advantages that enhance the performance of deep learning models in detecting underground defects using GPR data. Due to the constraints of the field test scale, GPR data from a single survey line were obtained for testing in the experiment. Given the limited dataset size, the prediction accuracy was compromised because the research focuses on the preprocessing of GPR data before the use of computer vision algorithms. The preprocessing methods, such as horizontal Sobel edge detection and Otsu’s thresholding, significantly improve the visibility of hyperbola features in GPR images. By enhancing the contrast and clarity of these features, the models can more effectively focus on the target regions, leading to higher detection accuracy. This is crucial because target features can be obscured by background noise and complex subsurface structures. In addition, preprocessing reduces the complexity of the data by minimizing the amount of noise for model training. By removing or reducing background noise, the models can converge faster with fewer computational resources. This speeds up the training process and reduces the risk of overfitting, resulting in better generalization of the hyperbolic feature detection algorithm. In this study, 39ns was selected because the depths of pavement defects were beyond 39ns. This time threshold may change according to different types of diseases. In addition, because the method relies on the dielectric contrast between internal defects and the surrounding soil—which varies with the moisture or air content inside the defects—detection outcomes could differ. Consequently, in practice, different radar configurations and weather conditions (e.g., raining, snowing, etc.) should be tested for validation in future study.

## 5. Conclusions

This study investigated the factors that could affect the hyperbola detectability of hidden defects in the asphalt pavement and proposed image processing methods to provide fast and straightforward visualization of GPR features. B-scan images from field tests were processed using methods such as edge detection, thresholding, histogram equalization, and clustering. A metric named RIHSNR was used to quantify the performance of hyperbolic feature detectability enhancement. The main conclusions are made in the following.

A horizontal Sobel detector is suggested to enhance hyperbolic features and remove stratified reflections during the GPR survey. Adaptive thresholding using default kernel size or Otsu’s thresholding is suggested after horizontal Sobel kernel detection to isolate hyperbolic reflection features based on RIHSNR scores. Based on RIHSNR scores and computational time, histogram equalization combined with horizontal Sobel and clustering is not recommended for hyperbolic feature visibility enhancement.

When combined with the proposed image processing methods (e.g., horizontal Sobel edge detection and Otsu’s thresholding), the performance of deep learning models (Faster R-CNN and CBAM-YOLOv8) was significantly enhanced in detecting subsurface objects using GPR data. The preprocessing techniques improved the visibility of hyperbola features and reduced noise, leading to notable optimizations in detection accuracy and efficiency. Specifically, the processed dataset achieved a higher mean average precision (mAP@0.5) compared with the raw dataset, with Faster R-CNN improving from 0.65 to 0.85 and CBAM-YOLOv8 improving from 0.72 to 0.88. The F1 score, which balances precision and recall, also showed significant gains, increasing from 0.68 to 0.82 for Faster R-CNN and from 0.75 to 0.89 for CBAM-YOLOv8. Additionally, the inference time was reduced, with Faster R-CNN decreasing from 45 ms to 30 ms and CBAM-YOLOv8 decreasing from 35 ms to 25 ms. These results demonstrate that integrating image processing with deep learning not only enhances detection accuracy but also improves computational efficiency, making it more suitable for real-time applications in complex environments.

The proposed method could help GPR operators identify subsurface objects during GPR tests in real-time. These methods are accessible and executable using the standard image processing libraries. The visualization of B-scan images using straightforward imaging processing methods will assist in fast and efficient determination of hidden defects in asphalt pavement using GPR.

## Figures and Tables

**Table 1:** High viscous asphalt characteristics.

Project		Unit	Test Results	Technical Indicators	Test Method
Zero shear viscosity at 60 °C		Pa·s	55,000	≥40,000	DG/TJ 08-2074
Dynamic viscosity at 60 °C		Pa·s	235,000	≥50,000	T0620
25 °C penetration		0.1 m	50	≥40	T0604
15 °C ductility		cm	98	≥80	T0605
5 °C ductility		cm	38	≥30	T0605
Softening point		°C	95.0	≥85	T0606
Flash point		°C	316	≥260	T0611
Solubility (trichloroethylene)		%	99.97	≥99	T0607
Elasticity recovery at 25 °C		%	98	≥90	T0662
25 °C viscosity		N·m	28.1	≥20	T0624
25 °C toughness		N·m	19.6	≥15	T0624
After TFOT	Mass changes	%	+0.050	≤0.6	T0609
	25 °C penetration ratio	%	90	≥70	T0604
	5 °C ductility	cm	18	≥15	T0605

**Table 2:** Aggregate density test results.

Type of Aggregate	Material Number	Relative Bulk Density	Bulk Density
Basalt	1	2.958	2.901
	2	2.906	2.843
	4	2.842	2.781
Limestone	1	2.719	2.684
	2	2.767	2.733
	3	2.753	2.715
	4	2.749	2.706
	5	2.760	2.674

**Table 3:** Marshall test results [41].

	Optimum Asphalt–Aggregate Ratio	Density	Void Content	VMA	Stability	VFA	Flow Value
	(%)	g/cm^3^	(%)	(%)	(KN)	(%)	(0.1 mm)
High-viscosity AC-20	4.3	2.464	3.3	12.1	13.0	73.2	32.3
Heavy-duty AC-20	4.2	2.477	3.9	11.6	14.3	74.9	31.9
Heavy-duty SMA-10	6.2	2.586	3.0	17.0	13.9	75.7	35.2
Heavy-duty SMA-13	5.8	2.609	4.4	17.3	14.2	76.2	37.1

**Table 4:** RIHSNR scores of adaptive thresholding using different kernel sizes.

Adaptive Thresholding Kernel Size	Score
3	31.48
5	46.01
7	62.76
9	78.80
Otsu’s thresholding	58.89
raw image	5.83

**Table 5:** RIHSNR scores of CLAHE using various clip limits.

Clip Limit	Score
0.01	35.55
0.02	21.84
0.03	15.79
0.04	12.55
0.05	10.49
Raw Image	5.83

**Table 6:** RIHSNR scores of K-means clustering with various K values.

K Value	Score
2	15.02
3	25.47
4	28.56
5	22.73
Raw image	5.83

**Table 7:** Comparisons of processing scores using raw GPR data and processed data based on preprocessing approaches.

Model	Dataset	mAP@0.5	F1 Score	Inference Time (ms)	Score
Faster R-CNN	Raw	0.65	0.68	45	12.44
	Preprocessed	0.85	0.82	30	16.65
CBAM-YOLOv8	Raw	0.72	0.75	30	27.84
	Preprocessed	0.88	0.89	25	33.54

## Data Availability

The original contributions presented in this study are included in the article. Further inquiries can be directed to the corresponding author.
